# PPIE in intervention studies: Randomized trials and clinical quality improvement

**DOI:** 10.1111/hex.13009

**Published:** 2020-01-24

**Authors:** Joseph W. LeMaster

**Affiliations:** ^1^ Department of Family Medicine and Community Health University of Kansas Medical Center Kansas City Missouri

Systematic reviews of randomized controlled trials and other intervention studies demonstrate that patient and public involvement and engagement in research studies promote cultural appropriateness; enhance recruitment; build community stakeholder capacity; assist teams in working through conflicts; and lead to higher quality outcome data and sustainability of the partnership after funding completes.^1^ Use of participatory groups may lead to more efficacious and cost‐effective outcomes.^2^

In this issue, a number of studies illustrate the benefits of PPIE at various stages throughout intervention studies.

Costello and Doris facilitated researchers and adolescents aged 10‐20 with rheumatic disease to co‐design a plain English research seminar for adolescents as well as a workshop about the scientific method. Before the seminar, researchers used a planning tool to identify gaps in their own PPI skill sets, identifying ‘comfort communicating with the target audience’. Their work improved researcher: adolescent communications and empowered adolescents to act as joint investigators. This led to calls from rheumatology researchers not involved in the study for further training in PPIE with young people, as well as from parents and young people themselves for expanded opportunities to collaborate.

Hayes‐Ryan et al conducted a prospective qualitative study embedded within a national, multi‐site randomized controlled trial of a diagnostic test for pre‐term, pre‐eclampsia in Ireland. Women were motivated to participate by both altruism, the potential of personnel benefit, that is potential earlier diagnosis of a clinical complication, and support by clinicians. Concern about the possibility for harm to their baby, however, was a barrier to participation. This emphasizes the importance of communicating risk clearly in a way that study participants can understand and assess prior to randomization in RCTs, so that participants who have strong opinions about the desirability of one intervention or its alternative are not disappointed if they then are randomized to a non‐preferred treatment group. If that occurs, participants are more likely to drop out or subvert the randomization, that is seek to participate in their preferred treatment.

Adario et al developed research guidelines integrating patients' voices into all stages of the development of patient‐reported outcome measures (PROs), including the identification of patient partners with the range of knowledge and experience needed to develop the PRO, clarify goals and tasks of PROs in language suitable for those with scientific backgrounds and develop governance structures including patients from start to finish.

Similarly, Skains et al conducted a cluster randomized trial of a shared decision‐making tool for use by clinicians and the parents of children seen in emergency departments for head trauma at intermediate risk of clinically significant brain injury. In a subgroup analysis, the decision aid, *Head CT Choice,* decreased decisional conflict and increased physician trust more in socioeconomically disadvantaged patients versus others. The investigators posited that clinician efforts to engage patients may have built their trust.

Pomey et al reported on the introduction of PPI into health technology assessments (HTA) conducted by the Canadian National Institute of Excellence in Health and Social Services (INESSS) that until their study conducted its assessments and developed guidance without patient involvement. Using a combination of participant observation, semi‐structured individual interviews and document review, patients living with an implantable cardiac defibrillators (ICD) shared their lived experience, co‐reviewed the literature to identify aspects of decision making and quality of life affecting care pathways for ICD patients and co‐authored the INESSS recommendations. This subsequently led INESSS decision‐makers to initiate a process to develop and apply a PPI framework in future HTA projects, as well as a new partnership between INESSS and the Center of Excellence on Partnership with Patients and the Public to train INESSS staff, patients and health‐care providers involved in HTA regarding PPI.

Campbell et al used a postal discrete choice experiment (DCE) among women who did not obtain cervical cancer screening after their first invitation during the STRATEGIC trial to identify their most preferred screening strategy. Similar to the trial, women responding (5.5% of those sent questionnaires) preferred home‐based self‐sampling kits. In the trial, however, women did not use the kits once they were sent to them, suggesting different concerns for actually using the kits than were hypothetically addressed in the DCE. This unique way to assess women's screening preferences compared to those used in the trial highlights the potential importance of women's awareness of consequences as drivers of real screening behaviour. The authors acknowledged the challenges of engaging this hard‐to‐reach population.

Selback et al (one investigator of who was a patient) interviewed 25 Dutch patients with lung cancer or chronic respiratory disease on the role of patient quality evaluations on physician performance, to support doctors' learning and assurance of their competence. Patient willingness to voice their concerns varied depending on their perceptions of power balance/dynamics in the doctor–patient relationship. The authors recommended providing ‘safe spaces’ for patients who feel more vulnerable to provide anonymous feedback regarding the quality of doctors’ care.

A number of other papers included in this issue observed care processes. These included direct observations of consultations between patients with dementia and memory care clinicians (Visser et al), and cancer patients and oncologists (Malhotra et al).

Others engaged patients regarding their personal care experience, including women with ductal carcinoma in situ (Nyhof et al), patients from culturally and linguistically diverse backgrounds (Harrison et al), patients at >10% risk of developing cardiovascular disease who received a relevant health check (Alageel et al) and Dutch patients re‐admitted to hospital within 30 days (Uitvlugt et al).

**Figure 1:**
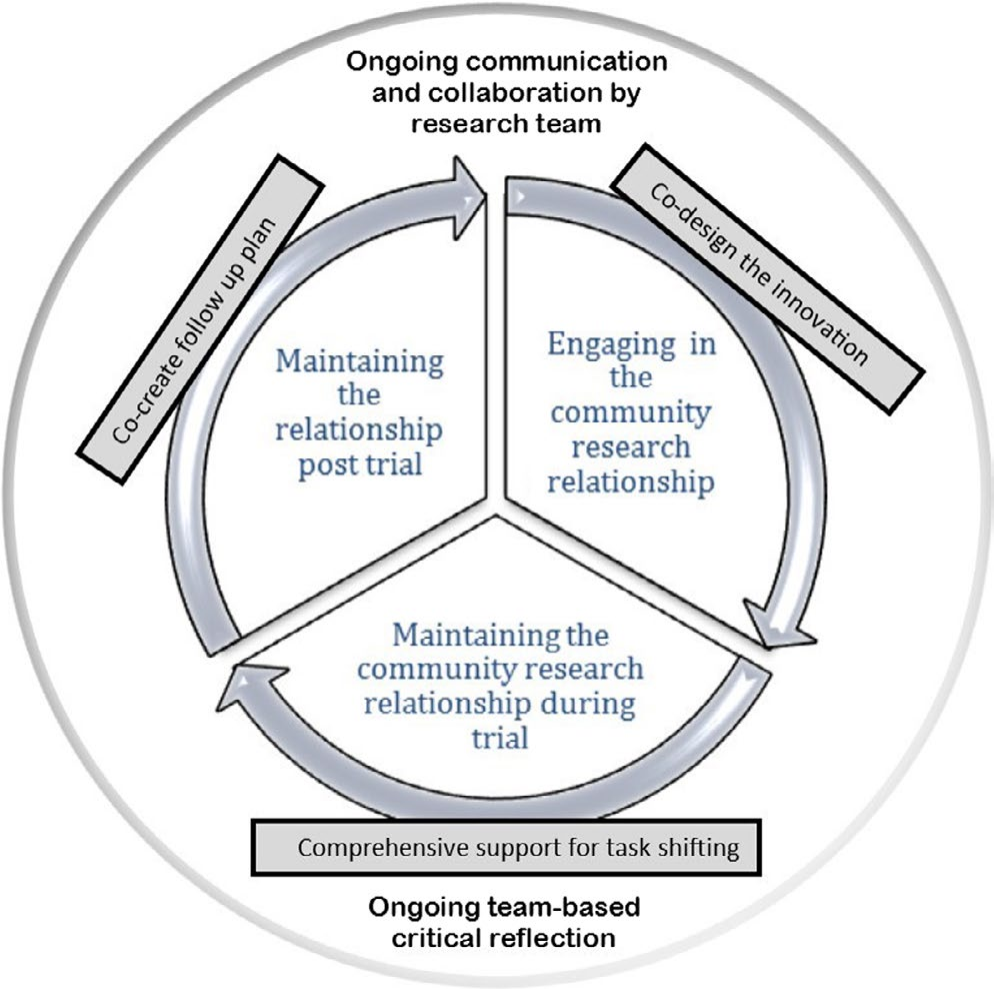
Community Engagement Cycle (Maar et al, used with permission)

PPIE in the analysis and dissemination of research studies remains an area for future studies to explore. Maar et al,^3^ in a recent patient‐engaged secondary analysis of intervention study data, identified culturally safe approaches to include in future RCTs with indigenous communities including the development of the intervention and its evaluation. The authors included rigorous member checks from community‐based indigenous co‐researchers on their thematic analytic team. They reported important relational elements of their approach that support PPI in intervention research (see Figure 1).

Given the above‐noted impacts, we recommend incorporating PPIE into all phases of future intervention and QI studies.
